# Extrafine Beclometasone Dipropionate/Formoterol NEXThaler on Device Usability, Adherence, Asthma Control and Quality of Life. A Panhellenic Prospective, Non-Interventional Observational Study in Patients with Asthma—The NEXT-Step Study

**DOI:** 10.3390/jpm12020146

**Published:** 2022-01-22

**Authors:** Petros Bakakos, Panagiotis Chatziapostolou, Panos Katerelos, Petros Efstathopoulos, Aliki Korkontzelou, Paraskevi Katsaounou

**Affiliations:** 1 1st University Department of Respiratory Medicine, Medical School, National and Kapodistrian University of Athens, “Sotiria” Chest Diseases Hospital, 11527 Athens, Greece; petros44@hotmail.com; 2 Pulmonary Clinic, Euromedica General Clinic of Thessaloniki, 54645 Thessaloniki, Greece; panoshatziapostolou@gmail.com; 3 BioStatistics PC, 11474 Athens, Greece; p.katerelos@bio-statistics.eu; 4 Chiesi Hellas S.A., 17455 Alimos, Greece; p.efstathopoulos@chiesi.com; 5 Pulmonary and Respiratory Failure Department, First ICU, Evangelismos Hospital, 10676 Athens, Greece; paraskevikatsaounou@gmail.com

**Keywords:** asthma, NEXThaler, usability, satisfaction, adherence, quality of life, inhalation device, extrafine, BDP/F

## Abstract

Background: The fixed combination of extrafine beclometasone dipropionate 100 μg/formoterol 6 μg (extrafine BDP/F) delivered by NEXThaler has proved to be effective in patients with moderate-to-severe asthma in terms of lung function, symptoms and asthma control. The aim of this study was to investigate the usability/satisfaction of NEXThaler and adherence to treatment in asthma patients not well controlled by low-dose inhaled corticosteroids (ICS). Methods: This was a 6-month prospective, multicenter, open-label, observational study in 661 patients with asthma not well controlled by low-dose ICS according to the physician’s clinical assessment, which have received regular treatment with extrafine BDP/F NEXThaler. Feeling of Satisfaction with Inhaler (FSI), treatment adherence with self-reported Morisky scale, asthma control, lung function and QoL were recorded at baseline, 3 and 6 months after treatment with extrafine BDP/F. Results: The percentage of patients at least “fairly” satisfied with NEXThaler usability (FSI-10 score 40 to 50) was 96.3%. The mean FSI-10 total score was 46.8 ± 4.4 on Visit 2 and increased to 48.1 ± 3.3 on Visit 3 (*p* < 0.001). Approximately 67% of the patients reported “high adherence” on Visit 2, and 70% of them reported “high adherence” on Visit 3. The percentage of patients with ACQ-6-uncontrolled asthma decreased from 79.1% on Visit 1 to 22.3% on Visit 2 and further decreased to 6.7% on Visit 3. Significant improvements were also observed in the total AQLQ score, predicted FEV1% and reduction in rescue medication use. Conclusions: The NEXThaler device, delivering a combination of BDP/F, achieves satisfaction and high adherence in patients with asthma not well controlled with low-dose ICS. Asthma control, QoL, lung function and rescue medication use were improved in a Greek real-world setting.

## 1. Introduction

Asthma is a heterogeneous chronic inflammatory disease of the airways that represents a major global public health issue affecting all age groups. Due to its increasing prevalence rates and rising treatment costs, it imposes an unacceptable burden to the patients and the community [1,2,3]. It is characterized by variable airflow obstruction, bronchial hyperresponsiveness and airway inflammation [1].

Regular controller treatment particularly with inhaled corticosteroid (ICS) containing medications markedly reduces the frequency and severity of asthma symptoms and the risk of having a flare up [1]. International guidelines recommend the combination of a long-acting beta-2-agonist (LABA) and an inhaled corticosteroid (ICS) when asthma is not fully controlled by low-dose ICS alone [1]. In the past several years, fixed dose combinations of ICS and LABA in a single inhaler have shown to improve adherence to asthma therapy [4]. Moreover, it has been shown that effectiveness and adherence to therapy are also related to the patient’s preference and attitude to a given device [5]. Preference of patients to receive either dry powder inhalers (DPIs) or metered dose inhalers (pMDIs) vary greatly. The use and clinical effectiveness rely on manual compliance, ability to synchronize pressing of the canister and inhaling slowly (pMDIs) or inhaling forcefully with sufficient inspiratory flow to overcome the resistance of the device (DPIs).

Recently, an additional delivery device option apart from pMDIs has been developed to provide the extrafine formulation of beclometasone dipropionate 100 μg/formoterol 6 μg (BDP/F) through a DPI, the NEXThaler. NEXThaler is a pocket-sized, breath-actuated, medium resistance, multi-dose DPI that proved to be an effective and well-tolerated delivery device for the treatment of patients with asthma who require maintenance treatment [5]. In a recent study, it was demonstrated that extrafine BDP/F delivered by NEXThaler was non-inferior to extrafine BDP/F administered via pMDI in terms of change from baseline in average pre-dose morning PEF and was superior over non-extrafine BDP-monotherapy [5]. Moreover, both BDP/F formulations (NEXThaler and pMDI) were comparable in terms of symptoms, rescue medication use and asthma control (evaluated by ACQ-7) [5].

The NEXThaler device has significant benefits compared to other DPIs, such as that dose counter does not decrement after preparation of the dose but only after delivery of the full therapeutic dose, acting like an “inhalation counter” [6]. The breath-actuated mechanism of the device is triggered by variable inhalation flows, and therefore, patients are able to use the device effectively irrespective of asthma control [6].

The aim of this study is to investigate the usability and satisfaction of NEXThaler delivering extrafine formulation of BDP/F and explore adherence to treatment, in patients with asthma not well controlled by low-dose ICS. Moreover, the improvement of asthma control with the use of BDP/F extrafine formulation and the quality of life will be assessed as well as lung function and use of rescue medication.

## 2. Materials and Methods

### 2.1. Participants

Patients were eligible if aged ≥18 and ≤75 years old with a physician-confirmed clinical diagnosis of persistent asthma according to GINA guidelines. They were all receiving low-dose ICS and, as needed, short-acting beta2 agonists (SABA) but had inadequate control of asthma according to the physician’s clinical assessment. A documented decision in the patient’s medical file on NEXThaler DPI BDP/F as the treatment of choice was needed before the patient being informed about their potential participation in the study.

Exclusion criteria included diagnosis of chronic obstructive pulmonary disease (COPD), cystic fibrosis, respiratory infection, or antibiotic intake the last 4 weeks before enrolment, other clinically significant medical conditions interfering with the patient’s compliance, pregnancy or lactating women. Moreover, patients who participated in interventional studies were excluded because their data do not reflect the standard clinical practice.

All patients were informed in detail about this study and gave their consent to the use of their data for processing and derivation of collective results.

### 2.2. Effectiveness Outcomes

The primary endpoint was the assessment of usability and patient satisfaction with the NEXThaler device. These were assessed using the FSI-10 (Feeling of Satisfaction with Inhaler) questionnaire. The FSI-10 questionnaire is a self-report instrument containing 10 questions, each with five possible responses on a five-point Likert scale: 5 = very, 4 = fairly, 3 = somewhat, 2 = not very, 1 = hardly at all [7]. It assesses the level of satisfaction of patients with the inhaler and includes items on ease or difficulty of use, portability and usability. The sum of the 10 individual scores represents the total FSI-10 score (minimum usability/satisfaction = 10, maximum usability/satisfaction = 50). The score of 40 corresponds on average to a “fairly” rating on the Likert scale, while a score of 50 corresponds to a “very” rating on the Likert scale. Accordingly, patients with total FSI-10 score between 40 and 50, represent patients who were at least “fairly” satisfied with the usability of the device [7]. This questionnaire has been translated and validated in a Greek population [8].

The co-primary endpoint was the evaluation of treatment adherence to BDP/F using the four-item Morisky Scale. The total score of the four-item Morisky Scale is the sum of the answers to the four items (minimum = 0, maximum = 4), with 0 points representing high adherence, 1–2 points representing intermediate adherence and 3–4 points representing low adherence [9].

Secondary outcomes were asthma control according to the six-item ACQ-6 questionnaire and quality of life as assessed by the AQLQ using the Greek abbreviated version of it. According to the ACQ score, asthma is classified as controlled when the ACQ score ≤ 0.75, partly controlled when 0.75 < ACQ score < 1.5 and uncontrolled when ACQ score ≥ 1.5 [10]. The Greek abbreviated version of the questionnaire AQLQ consists of 18 items of four domains: symptoms, activity limitation, sleep and environmental stimuli. The patient rates each one of the 18 items by using a seven-point scale, where “1” represents the absence of disturbance/limitation, while “7” the excessive disturbance/limitation. The mean score of the answers of each domain represents the domain score, while the mean score of the 18 questions represents the total questionnaire score [11]. Moreover, other secondary endpoints were a change in the predicted FEV1% and use of rescue medication (puffs/week of SABA as needed) compared to baseline.

### 2.3. Study Design

This was a multicenter, non-interventional, prospective, open label, observational study of recording and analyzing data from patients with asthma receiving a BDP/F NEXThaler according to the standard clinical practice for managing inadequately controlled or uncontrolled asthma by low-dose ICS plus SABA as needed. In total, sixty-two (62) private care sites participated in this study, coordinated by one hospital/site. This study was conducted as per the guidelines of the Declaration of Helsinki and was approved by the Institutional Review Board of the coordinating hospital.

Data were collected from December 2015 to January 2017 and chronologically covered 6 months, from the commencement of the treatment with BDP/F (baseline, Visit 1) and 3 and 6 months following patients’ enrolment and initiation of the study (Visit 2 and 3, respectively). Patients’ demographic and social data were collected at visit 1, including gender, age, family history, smoking status and height and weight in order to calculate BMI at the phase of statistical analysis. Medical history data on comorbidities were collected, with an emphasis on atopy and chronic rhinitis. During every visit, the following data were collected: treatment with study medication, usability satisfaction with the device (FSI-10), evaluation of treatment compliance (Morisky scale), asthma control (ACQ-6), asthma quality of life (AQLQ), lung function test results (if spirometry data were available), rescue medication use and other concomitant medications.

### 2.4. Statistical Methods

Analysis included all patients who gave their informed consent and met the inclusion criteria. Continuous parameters were presented using mean ± standard deviation, median, while the nominal ones (e.g., gender) were presented using tables of frequencies. The ordinal study parameters were presented using both descriptive measures (mean, standard deviation, etc.) and tables of frequencies.

The percentage of patients with a total FSI-10 score on the 3rd Visit between 40 and 50 (≥40 and ≤50) was calculated. All Confidence Intervals (CIs) to be calculated were of normal approximation, 95%, two-sided, and for all statistical tests: α = 0.05. Regarding the primary study variables, a 95% CI was computed for the estimation of the percentage of patients with total FSI-10 score on the 3rd Visit between 40 and 50 (≥40 and ≤50). For each primary variable, a *t*-test was used to compare the corresponding mean values of the two study visits.

Multiple linear regression (stepwise selection) tested the relationship of the average FSI-10 score (dependent variable) with the main individual data and average Morisky score (independent variables). Multiple linear regression (stepwise selection) also tested the relationship of the average Morisky scale (dependent variable) with the main individual data and average FSI-10 score (independent variables).

Regarding the secondary study variables, a multiple linear regression (stepwise selection) tested the relationship between the baseline values of each secondary variable (dependent variable), with the main individual data (independent variables). Repeated measures analysis of variance tested the change of each secondary variable over time. Paired samples *t*-test was applied for the (three) pairwise comparisons between study visits.

Friedman’s technique tested the change of ACQ-6 asthma classification over time. A Wilcoxon test was used for the (three) pairwise comparisons between visits. Paired *t*-test samples were applied for the pairwise comparisons between the mean scores of the four AQLQ domains. Regarding ACQ-6 and AQLQ differences between the 1st and the 3rd Visit (dependent variables) multiple linear regression (stepwise selection) was applied to test the relationship of these differences, with main demographic data, average Morisky score, average FSI-10 score and baseline values of the corresponding dependent variable.

## 3. Results

A total of 661 patients with asthma participated in the study, in a recruitment period that lasted 7 months. Only five patients (0.8%) prematurely discontinued treatment. The main demographic data and characteristics of the study group are shown in Table 1. A total of 486 patients (73,5%) received constant dosage of BDP/F during the whole study, while 387 patients (79.6%) were receiving two inhalations twice daily throughout the study.

### 3.1. FSI-10

**Figure 1:**
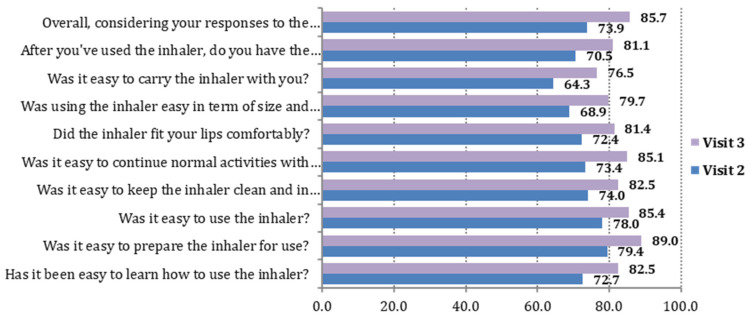
Visits 2 and 3. The 10 items of the FSI-10 Questionnaire: percentage (%) of patients who answered “very”.

The mean value of FSI-10 total score was 46.8 ± 4.4 on Visit 2 and 48.1 ± 3.3 on Visit 3 (an increase of 1.3, *p* < 0.001). The percentage of patients who were at least “fairly” satisfied with the usability of the NEXThaler device (FSI-10 score between 40 and 50) was 96.3% (95% C.I. 94.9%, 97.8%) on Visit 3. In all 10 questions of the FSI-10 Questionnaire, the percentage of patients who answered “very” increased in Visit 3 compared to Visit 2 by approximately 10%. Moreover, the median value of all items of the FSI-10 Questionnaire was 5.0 (“very”) at both Visits 2 and 3 (Figure 1).

The item with the higher “very” percentage, rating 5 out of 5 on the FSI scale, for both study visits was “Was it easy to prepare the inhaler for use?” (corresponding percentages on Visits 2 and 3: 79.4% and 89.0%). The items with the lower “very” percentages for both study visits were “Was it easy to carry the inhaler with you?” (corresponding percentages on Visits 2 and 3: 64.3% and 76.5%) and “Was using the inhaler easy in term of size and weight?” (corresponding percentages on Visits 2 and 3: 68.9% and 79.7%) (Table 2).

According to Multiple Linear Regression, Morisky scale and age were significantly related with the average total FSI-10 score. Patients with a lower Morisky scale (greater compliance) and younger patients showed a higher FSI-10 total score (see Supplementary Table S1).

### 3.2. Morisky Scale

Approximately 67% of the patients reported “high adherence” on Visit 2, and 70% of the patients reported “high adherence” on Visit 3. The mean value of the total score on the Morisky scale was 0.6 ± 1.0 on Visit 2 and 0.6 ± 1.1 on Visit 3 (*p* = 0.937) (Table 3). It must also be noted that the median value of the total score on the Morisky scale was 0.0 (“high adherence”) for both Visits 2 and 3. 

According to Multiple Linear Regression, the average FSI-10 total score, age and ACQ-6 score were significantly related with the total average score on the Morisky scale. Patients with a higher total FSI-10 score, older patients and patients with a higher baseline ACQ-6 score (less controlled) showed a lower total Morisky scale score (higher adherence) (see Supplementary Table S2).

### 3.3. Asthma Control (ACQ-6)

**Figure 2:**
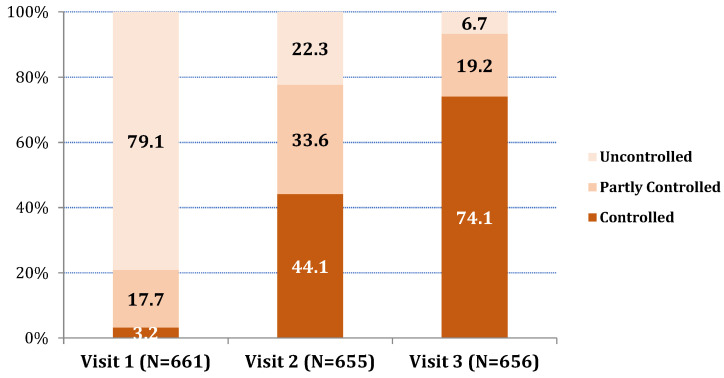
ACQ-6 classification (%) for the three visits of the study.

The mean ACQ-6 values decreased at Visit 2 in comparison to baseline (Visit 1) and further decreased on Visit 3 (Hotelling’s test *p* < 0.001 and *t*-test *p* < 0.001 for all pair comparisons between the three visits). The mean ACQ-6 values decreased from 2.3 on Visit 1 to 0.9 on Visit 2 and 0.5 on Visit 3 (average decrease (±SD) on Visit 3 compared to Visit 1 = 1.8 ± 1.0). The percentage of patients with ACQ-6-uncontrolled asthma decreased from 79.1% on Visit 1 to 22.3% on Visit 2 and further decreased to 6.7% on Visit 3. Accordingly, the percentage of patients with ACQ-6-controlled asthma increased from 3.2% on Visit 1 to 44.1% on Visit 2 and further increased to 74.1% on Visit 3 (Friedman’s test *p* < 0.001 and Wilcoxon test *p* < 0.001 for all pair comparisons between the three visits) (Figure 2). According to Multiple Linear Regression the variables detected as significantly related to ACQ-6 differences between Visit 1 and 3 (Visit 1 minus Visit 3) were baseline values of ACQ-6 (patients with higher baseline ACQ-6 values showed a greater ACQ-6 decrease) and FSI-10 average total score (patients with higher FSI-10 values showed a greater ACQ-6 decrease) (see Supplementary Table S3).

### 3.4. Quality of Life (AQLQ)

**Figure 3:**
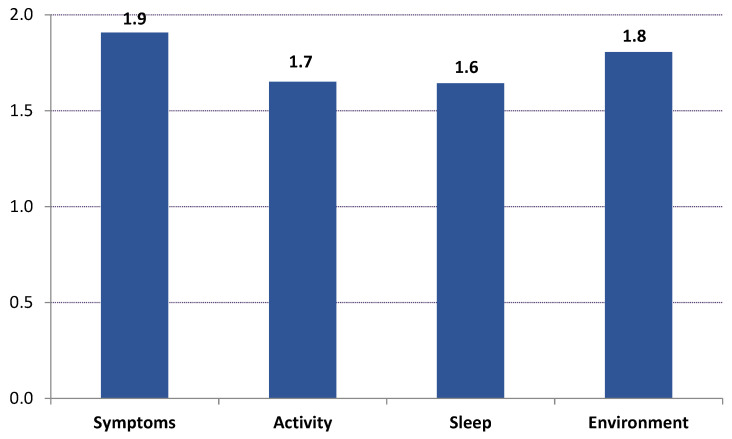
Mean values of the differences between Visit 1 and Visit 3 (Visit 3 minus Visit 1 values) for each AQLQ Domain.

The mean (and median) values of all 18 AQLQ items were increased in Visits 2 and 3 compared to baseline. The mean score of each of the four domains of AQLQ and the mean total score increased substantially on Visit 2 compared to Visit 1, and further increased on Visit 3 (Hotelling’s test *p* < 0.001, and *t*-test *p* < 0.001 for all pair comparisons between the three visits). The mean AQLQ total score increased from 4.6 on Visit 1, to 5.9 on Visit 2 and 6.4 on Visit 3 (average increase on Visit 3 compared to Visit 1 = 1.8 ± 1.1). The Domains “Symptoms” and “Environment” presented the largest increase (Figure 3). According to Multiple Linear Regression, the variables detected as significantly related with AQLQ differences between Visit 1 and Visit 3 (Visit 1 minus Visit 3) were baseline values of AQLQ (patients with lower baseline AQLQ values showed a greater AQLQ increase) and FSI-10 average total score (patients with higher FSI-10 values showed a greater AQLQ increase) (see Supplementary Table S4).

### 3.5. Lung Function

FEV1 was recorded in 338 patients during the 1st Visit, in 238 patients during the 2nd and in 233 patients during the 3rd Visit. The mean predicted FEV1% and FVC% predicted increased on Visit 2 compared to Visit 1 and further increased on Visit 3 (Hotelling’s test *p* < 0.001, and *t*-test *p* < 0.001 for all pair comparisons between the three visits). The mean predicted FEV1% increased from 80.7% on Visit 1 to 87.0% on Visit 2 and 88.5% on Visit 3 (average increase on Visit 3 compared to Visit 1 = 9.7 ± 12.5) (see Supplementary Figure S1). Mean FEV1 increased from 2587.2 mL on the 1st Visit, to 2686.6 mL on the 2nd Visit and 2789.6 mL on the 3rd one (repeated measures analysis of variance, Greenhouse–Geisser statistic <0.001 and *t*-test *p*-value < 0.001 for all pair comparisons between the three visits) (see Supplementary Figure S2). The average increase on the 3rd Visit compared to the 1st Visit was 275.7(±424.4) mL. The mean FVC% predicted increased from 86.0% on Visit 1, to 89.3% on Visit 2 and 90.1% on Visit 3 (average increase on Visit 3 compared to Visit 1 = 6.8 ± 12.8) (see Supplementary Figure S3).

### 3.6. Rescue Medication

**Figure 4:**
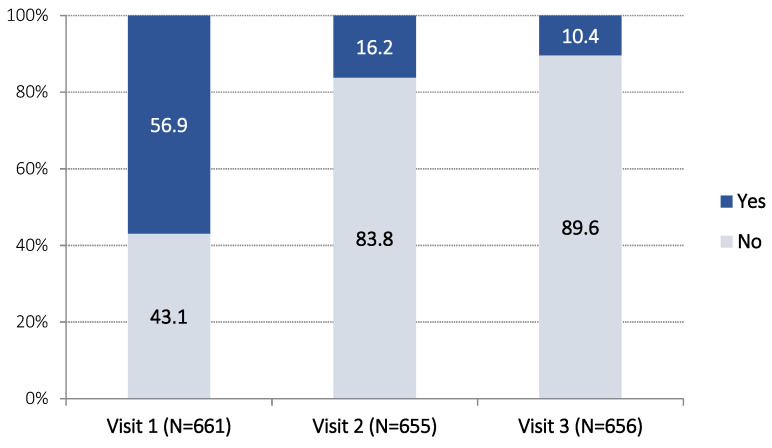
Percentage of patients using short-acting beta-2 agonists (yes-no), during the last month before each visit.

The percentage of patients who used short-acting beta-2 agonists (the last month before each Visit) decreased over time. During Visit 1, the corresponding percentage was 56.9%, during Visit 2 this was 16.2%, while during Visit 3 this was only 10.4% (Figure 4). During Visit 1, the mean (±SD) number of times of weekly use of short-acting beta-2 agonists (the ast month before each Visit) was 6.0 ± 10.5, during Visit 2 this was 1.0 ± 3.5, while during Visit 3 this was only 0.6 ± 3.1 times per week (Hotelling’s test *p* < 0.001. *t*-test *p* < 0.001 for the pairs Visit 1-Visit 2 and Visit 1-Visit 3, *p* = 0.002 for the pair Visit 2-Visit 3). 

## 4. Discussion

In the present study, we evaluated the usability and patient satisfaction of the NEXThaler device delivering the inhaled combination of BDP/F extrafine formulation in patients with asthma not well controlled by low-dose ICS. We found that the percentage of patients who were at least “fairly” satisfied with the usability of the NEXThaler device presenting an FSI-10 score between 40 and 50 surpassed96%. Furthermore, the usability/satisfaction was indicated by the median value of 5 corresponding to “very” in each of the 10 individual items of the FSI score. Moreover, the adherence to treatment was quite high, and almost 70% of the patients with asthma presented high adherence after 3 and 6 months of treatment. Furthermore, we showed that this ICS/LABA combination is very effective in improving the level of asthma control, quality of life and lung function in patients not well controlled on low-dose ICS monotherapy and also led to lesser use of rescue medication (SABA).

Fixed combinations of ICS/LABA are considered convenient and are the mainstay of asthma treatment. They are available in many different formulations and devices and have helped in achieving better compliance and better control of the disease. Not all patients with asthma are equally capable of using a particular device that delivers the inhaled combination. Therefore, the availability of the same medication in different formulations, such as pMDIs and DPIs, offers both physicians and patients a broader range of therapeutic options to treat asthma and improve compliance.

An additional delivery device option apart from pMDI has been developed to provide the extrafine formulation of BDP/F through a DPI, the NEXThaler. NEXThaler was proved to be an effective and well-tolerated delivery device for the treatment of patients with asthma who require regular treatment with ICS/LABA and was shown to be non-inferior to extrafine BDP/F administered via pMDI in terms of lung function, asthma control and use of rescue medication [5].

Personalized medicine, having a broader meaning than precision medicine, includes all features associated with “the person” [12]. In this context, satisfaction and preference for a device, encompassing functional, emotional and psychological aspects, can be perceived as an essential component of personalized management in asthma treatment. In a study comparing the effectiveness and satisfaction of different DPIs (NEXThaler, Turbuhaler, Diskus), NEXThaler was found to be superior to the other two DPIs in terms of the number of device use failures, which were significantly less, the time to set up, which was quicker, and time to read the leaflet, which was faster. Patients rated the NEXThaler DPI as the easiest to use and the most preferred inhaler [13]. Accordingly, the patient’s level of adherence to treatment was increased. This is in accordance with our findings, where high satisfaction and usability of the NEXThaler was associated with high adherence to treatment.

NEXThaler device has significant advantages such as that the dose counter works as an “inhalation counter”, decrementing only after delivery of the full therapeutic dose, but most importantly, the breath-actuated mechanism of the device is triggered by variable inhalation flows. That provides the opportunity to the patients to use the device effectively irrespective of asthma control and severity [6].

The self-reported Morisky Scale has been used in many studies to assess the adherence to treatment among patients with asthma [14,15]. We found that more than 2/3 of our patients reported high adherence after 6 months of treatment with a BDP/F NEXThaler.

A correct inhaler technique is the key factor to ensure that the inhaled drug reaches the bronchial tree, exerts its pharmacological effects and, therefore, improves asthma control. Asthma control is the main target of asthma care and pharmacotherapy according to asthma guidelines [1]. We found that BDP/F delivered by NEXThaler improves asthma control significantly by increasing the percentage of patients with ACQ-6-controlled asthma from 3.2% to 74.1% after a 6-month treatment. Lung function also significantly improved, as shown by the increase of FEV1 (% predicted and in mL) and FVC% predicted. Importantly, in terms of FEV1, the change from baseline exceeded 270 mL. It should be mentioned that no minimal clinically important difference has been clearly established yet for FEV1 in asthma patients. Different cut-offs have been proposed in the literature, ranging from 100 mL in patients with COPD [16] to 230 mL in patients with asthma [17], which our results outperformed. These findings are in accordance with clinical trials that showed the efficacy of the combination BDP/F in improving lung function and asthma control either delivered by pMDI or by NEXThaler [5,18,19].

The improvement in quality of life observed in our study accompanying the improvement in asthma control is compatible with other studies showing a positive correlation between asthma control and quality of life [20]. It is worth mentioning that both ACQ and AQLQ showed clinically relevant changes. ACQ and AQLQ scores showed a drop of 1.8 units between Visits 1 and 3, far exceeding the threshold of the minimal important difference of 0.5, meaning that such changes will be perceived by patients as beneficial [21,22].

NEXThaler is substantially unaffected by flow rate through the inhaler in terms of both delivered dose and fine particle mass [23], and this may partly explain the satisfaction of patients with asthma with the device since the effect is not influenced by the applied flow rate.

Inhaler device selection can have an impact on asthma clinical outcomes and the use of healthcare resources. Although we did not assess the use of healthcare resources in the present study, we showed that BDP/F NEXThaler improved all outcomes after 6 months of treatment in patients with asthma not well controlled by low-dose ICS. This could be attributed to the high satisfaction/usability and high adherence achieved by the NEXThaler device and in every day clinical practice the latter is of great significance in attaining better clinical outcomes. Importantly, our study showed that higher FSI scores correlated with lower Morisky scale scores, meaning greater compliance, in the Greek population studied. Our results seem to show cohesion with other studies reporting positive correlations between patients’ satisfaction, treatment adherence and clinical outcomes [24,25].

Our study was conducted as per GINA 2015 recommendations [26], and patients were included in the study if not well controlled in step 2 treatment with low-dose ICS. At that time point, the only recommended reliever medication for step 2 was SABA. After stepping up to step 3, there were two options for reliever medication: SABAs or ICS/formoterol. However, in order to have a more homogenous population, and since the primary outcome was patients’ satisfaction, we preferred to keep SABAs as a reliever and evaluate rescue medication as a secondary outcome. Importantly, subsequent studies established the use of low-dose ICS/formoterol combinations as maintenance and reliever treatment as the preferred option in step 3 [1], thus further raising “the device” into a high on the list factor so as to achieve the maximum therapeutic potential.

In terms of study limitations, it must be highlighted that a step-up approach was followed during this study in terms of escalating asthma treatment according to GINA recommendations and everyday clinical practice, which could have an impact on asthma control. Furthermore, rescue medication was assessed by the number of puffs/week of SABA needed by the patient during the last month before each visit. We acknowledge the use of diaries or e-diaries in assessing such clinical outcomes; nevertheless, we consider that the study population could have successfully recalled any use in the recent past.

In conclusion, we showed that the fixed combination of BDP/F delivered by NEXThaler is a rational choice for the treatment of patients with asthma who feel very satisfied with the device and present high adherence, while being at the same time very effective in clinical outcomes such as asthma control, quality of life, lung function and use of rescue medication.

## Figures and Tables

**Table 1:** Main demographic data and characteristics of the study group (N = 661).

Main Demographic Data and Characteristics		Ν	%
Gender:	Female	397	60.1
	Male	264	39.9
Age (years)	<45	274	41.5
	45–64	258	39.0
	≥65	129	19.5
BMI classification	Underweight	8	1.2
	Normal	258	39.0
	Overweight	233	35.2
	Obese	162	24.5
Family history of asthma	No	442	66.9
	Yes	219	33.1
Smoking	No	469	71.0
	Yes	192	29.0
History of atopy	No	418	63.2
	Yes	243	36.8
Chronic rhinitis	No	365	55.2
	Yes	296	44.8
	Total	661	100.0

BMI: Body Mass Index.

**Table 2:** FSI-10 Questionnaire: distribution of each of the 10 items during the two visits after baseline.

		Visit 2		Visit 3	
		N	%	N	%
1. Has it been easy to learn how to use the inhaler?	hardly at all	1	0.2	0	0.0
	not very	0	0.0	0	0.0
	somewhat	32	4.9	14	2.1
	fairly	146	22.3	101	15.4
	very	476	72.7	541	82.5
2. Was it easy to prepare the inhaler for use?	hardly at all	1	0.2	0	0.0
	not very	0	0.0	0	0.0
	somewhat	21	3.2	8	1.2
	fairly	112	17.1	64	9.8
	very	521	79.5	584	89.0
3. Was it easy to use the inhaler?	hardly at all	1	0.2	0	0.0
	not very	1	0.2	0	0.0
	somewhat	15	2.3	8	1.2
	fairly	126	19.2	88	13.4
	very	512	78.2	560	85.4
4. Was it easy to keep the inhaler clean and in good working condition?	hardly at all	1	0.2	0	0.0
	not very	1	0.2	0	0.0
	somewhat	10	1.5	12	1.8
	fairly	158	24.1	103	15.7
	very	485	74.0	541	82.5
5. Was it easy to continue normal activities with the use of inhaler?	hardly at all	1	0.2	0	0.0
	not very	1	0.2	0	0.0
	somewhat	21	3.2	11	1.7
	fairly	152	23.2	87	13.3
	very	480	73.3	558	85.1
6. Did the inhaler fit your lips comfortably?	hardly at all	1	0.2	0	0.0
	not very	2	0.3	1	0.2
	somewhat	30	4.6	14	2.1
	fairly	149	22.7	107	16.3
	very	473	72.2	534	81.4
7. Was using the inhaler easy in term of size and weight?	hardly at all	1	0.2	0	0.0
	not very	2	0.3	1	0.2
	somewhat	40	6.1	19	2.9
	fairly	161	24.6	113	17.2
	very	451	68.9	523	79.7
8. Was it easy to carry the inhaler with you?	hardly at all	1	0.2	0	0.0
	not very	12	1.8	4	0.6
	somewhat	59	9.0	28	4.3
	fairly	162	24.7	122	18.6
	very	421	64.3	502	76.5
9. After you’ve used the inhaler, do you have the feeling that you used it correctly?	hardly at all	1	0.2	0	0.0
	not very	0	0.0	1	0.2
	somewhat	20	3.1	6	0.9
	fairly	173	26.4	117	17.8
	very	461	70.4	532	81.1
10. Overall, considering your responses to the previous questions, were you satisfied	hardly at all	1	0.2	0	0.0
	not very	0	0.0	0	0.0
	somewhat	4	0.6	1	0.2
	fairly	167	25.5	93	14.2
	very	483	73.7	562	85.7
	Total	655	100.0	656	100.0

**Table 3:** Morisky scale: distribution of each of the four items during the two visits after baseline.

		Visit 2		Visit 3	
		N	%	N	%
Do you ever forget to take your medicine?	No	524	80.0	514	78.4
	Yes	131	20.0	142	21.6
Are you careless at times about taking your medicine?	No	526	80.3	524	79.9
	Yes	129	19.7	132	20.1
When you feel better do you sometimes stop taking your medicine?	No	535	81.7	545	83.1
	Yes	120	18.3	111	16.9
Sometimes if you feel worse when you take the medicine, do you stop taking it?	No	629	96.0	629	95.9
	Yes	26	4.0	27	4.1
	Total	655	100.0	656	100.0

## Data Availability

The data presented in this study are available on request from the corresponding author. Chiesi Hellas S.A. commits to sharing with qualified scientific and medical researchers, conducting legitimate research, the anonymized patient-level and study-level data, the clinical protocol and the full clinical study report of Chiesi Hellas-sponsored non-interventional clinical trials.

## Supplementary Materials

The following are available online at https://www.mdpi.com/article/10.3390/jpm12020146/s1, Figure S1: FEV1% predicted/Mean ± SD on the 3 Visits of the study. Figure S2: FEV1 (mL) Mean ± SD on the 3 Visits of the study. Figure S3: FVC% predicted/Mean ± SD on the 3 Visits of the study. Table S1: Multiple Linear Regression analysis. Dependent variable: FSI-10 total score, average values of the two Visits., Table S2: Multiple Linear Regression analysis. Dependent variable: Morisky scale, average values of the two Visits., Table S3: Multiple Linear Regression analysis. Dependent variable: ACQ-6., Table S4: Multiple Linear Regression analysis. Dependent variable: AQLQ.

## References

[1] Global Initiative fοr Asthma Global Strategy for Asthma Management and Prevention www.gina.org 2021. https://ginasthma.org/gina-reports/.

[2] Masoli M., Fabian D., Holt S., Beasley R., Global Initiative for Asthma (GINA) Program (2004). The global burden of asthma: Executive summary of the GINA Dissemination Committee Report. Allergy.

[3] Barnes P.J., Jonsson B., Klim J.B. (1996). The costs of asthma. Eur. Respir. J..

[4] Chapman K.R., Barnes N.C., Greening A.P., Jones P.W., Pedersen S. (2010). Single maintenance and reliever therapy (SMART) of asthma: A critical appraisal. Thorax.

[5] Kanniess F., Scuri M., Vezzoli S., Francisco C., Petruzzelli S. (2015). Extrafine beclomethasone/formoterol combination via a dry powder inhaler (NEXThaler^®^) or pMDI and beclomethasone monotherapy for maintenance of asthma control in adult patients: A randomised, double-blind trial. Pulm. Pharmacol. Ther..

[6] Corradi M., Chrystyn H., Cosio B.G., Pirozynski M., Loukides S., Louis R., Spinola M., Usmani O.S. (2014). NEXThaler, an innovative dry powder inhaler delivering an extrafine fixed combination of beclometasone and formoterol to treat large and small airways in asthma. Expert Opin. Drug Deliv..

[7] Torderaa M.P., Viejob J.L., Sanchisc J., Badiad X., Cobose N., Picadof C., Sobradillog V., del Ríoh J.M.G., Ducei F., Cabreraj L.M. (2008). Satisfacción y preferencia del paciente asmático por los dispositivos de inhalación. Aplicación del FSI-10 [Assessment of patient satisfaction and preferences with inhalers in asthma with the FSI-10 Questionnaire]. Arch. Bronconeumol..

[8] Grekas N., Athanassiou K., Papataxiarchou K., Porichi O., Perpina-Tordera M. (2011). Reliability of the FSI-10 questionnaire for the assessment of of the usability of drug inhalers in Greek patients. Arch. Hellenic. Med..

[9] Choi T.N., Westermann H., Sayles W., Mancuso C.A., Charlson M.E. (2008). Beliefs About Asthma Medications: Patients Perceive Both Benefits and Drawbacks. J. Asthma.

[10] Juniper E., O′byrne P., Guyatt G., Ferrie P., King D. (1999). Development and validation of a questionnaire to measure asthma control. Eur. Respir. J..

[11] Juniper E.F., Guyatt G.H., Epstein R.S., Ferrie P.J., Jaeschke R., Hiller T.K. (1992). Evaluation of impairment of health related quality of life in asthma: Development of a questionnaire for use in clinical trials. Thorax.

[12] Canonica G.W., Ferrando M., Baiardini I., Puggioni F., Racca F., Passalacqua G., Heffler E. (2018). Asthma: Personalized and precision medicine. Curr. Opin. Allergy Clin. Immunol..

[13] Voshaar T., Spinola M., Linnane P., Campanini A., Lock D., LaFratta A., Scuri M., Ronca B., Melani A.S. (2014). Comparing Usability of NEXThaler^®^ with Other Inhaled Corticosteroid/Long-Acting β2-Agonist Fixed Combination Dry Powder Inhalers in Asthma Patients. J. Aerosol Med. Pulm. Drug Deliv..

[14] Guénette L., Breton M.-C., Grégoire J.-P., Jobin M.-S., Bolduc Y., Boulet L.-P., Dorval E., Moisan J. (2015). Effectiveness of an asthma integrated care program on asthma control and adherence to inhaled corticosteroids. J. Asthma.

[15] Unni E., Shiyanbola O.O. (2015). Clustering medication adherence behavior based on beliefs in medicines and illness perceptions in patients taking asthma maintenance medications. Curr. Med. Res. Opin..

[16] Cazzola M., MacNee W., Martinez F.J., Rabe K.F., Franciosi L.G., Barnes P.J., Brusasco V., Burge P.S., Calverley P.M.A., Celli B.R. (2008). Outcomes for COPD pharmacological trials: From lung function to biomarkers. Eur. Respir. J..

[17] Santanello N., Zhang J., Seidenberg B., Reiss T., Barber B. (1999). What are minimal important changes for asthma measures in a clinical trial?. Eur. Respir. J..

[18] Dhillon S., Keating G.M. (2006). Beclometasone Dipropionate/Formoterol. Drugs.

[19] Huchon G., Magnussen H., Chuchalin A., Dymek L., Gonod F.B., Bousquet J. (2009). Lung function and asthma control with beclomethasone and formoterol in a single inhaler. Respir. Med..

[20] Bateman E.D., Bousquet J., Keech M.L., Busse W.W., Clark T.J.H., Pedersen S.E. (2006). The correlation between asthma control and health status: The GOAL study. Eur. Respir. J..

[21] Bonini M., Di Paolo M., Bagnasco D., Baiardini I., Braido F., Caminati M., Carpagnano E., Contoli M., Corsico A.G., Del Giacco S. (2020). Minimal clinically important difference for asthma endpoints: An expert consensus report. Eur. Respir. Rev..

[22] Juniper E.F. (1994). Determining a minimal important change in a disease-specific quality of life questionnaire. J. Clin. Epidemiology.

[23] Buttini F., Brambilla G., Copelli D., Sisti V., Balducci A.G., Bettini R., Pasquali I. (2016). Effect of Flow Rate on In Vitro Aerodynamic Performance of NEXThaler^®^ in Comparison with Diskus^®^ and Turbohaler^®^ Dry Powder Inhalers. J. Aerosol Med. Pulm. Drug Deliv..

[24] Small M., Anderson P., Vickers A., Kay S., Fermer S. (2011). Importance of inhaler-device satisfaction in asthma treatment: Real-world observations of physician-observed compliance and clinical/patient-reported outcomes. Adv. Ther..

[25] Mäkelä M.J., Backer V., Hedegaard M., Larsson K. (2013). Adherence to inhaled therapies, health outcomes and costs in patients with asthma and COPD. Respir. Med..

[26] Global Initiative fοr Asthma Global Strategy for Asthma Management and Prevention www.gina.org 2015. https://ginasthma.org/archived-reports/.

