# Supplementary material for: Extrafine Beclometasone Dipropionate/Formoterol NEXThaler on Device Usability, Adherence, Asthma Control and Quality of Life. A Panhellenic Prospective, Non-Interventional Observational Study in Patients with Asthma—The NEXT-Step Study
Source: J Pers Med. 2022 Jan 22;12(2):146. doi: 10.3390/jpm12020146 (PMC8876660; doi:10.3390/jpm12020146)
Supplement: Supplementary file 1 [file jpm-12-00146-s001.zip › jpm-1459874-supplementary.pdf]

Journal of Personalized Medicine

SUPPLEMENTARY APPENDIX

EXTRAFINE BECLOMETASONE DIPROPIONATE/ FORMOTEROL NEXTHALER ON USABILITY OF DEVICE, ADHERENCE, ASTHMA CONTROL AND QUALITY OF LIFE. A PANHELLENIC PROSPECTIVE, NON-INTERVENTIONAL OBSERVATIONAL STUDY IN PATIENTS WITH ASTHMA - THE NEXT-STEP STUDY

Petros Bakakos[1], Panagiotis Chatziapostolou[2], Panos Katerelos[3], Petros Efstathopoulos[4], Aliki Korkontzelou[5] and Paraskevi Katsaounou[6]

[1] 1st University Department of Respiratory Medicine, National and Kapodistrian University of Athens, Medical School, "Sotiria" Chest Diseases Hospital, 11527 Athens, Greece; petros44@hotmail.com
[2] Pulmonary Clinic, Euromedica General Clinic of Thessaloniki, 54645 Thessaloniki, Greece; panoshatziapostolou@gmail.com
[3] BioStatistics PC; p.katerelos@bio-statistics.eu
[4] Chiesi Hellas S.A.; p.efstathopoulos@chiesi.com
[5] Chiesi Hellas S.A.; a.korkontzelou@chiesi.com
[6] Pulmonary and Respiratory Failure Dpt, First ICU, Evangelismos Hospital, Ipsilandou 45-7, 10676 Athens, Greece; paraskevikatsaounou@gmail.com
* Correspondence: Korkontzelou Aliki; a.korkontzelou@chiesi.com

3.1. FSI-10

Adjusted R^2= 0.212	Unstandardized Coefficients		Standardized Coefficients	t-statistic	p-value
	B Coefficient	S.E.	Beta Coefficient		
Constant	50.050	0.435		115.150	<0.001
Average MORISKY scale	-1.467	0.127	-0.403	-11.570	<0.001
Age (years)	-0.041	0.008	-0.184	-5.209	<0.001

Table 1 SA. Multiple Linear Regression analysis. Dependent variable: FSI-10 total score, average values of the two Visits.

3.2 Morisky scale

Adjusted R^2= 0.181	Unstandardized Coefficients		Standardized Coefficients	t-statistic	p-value
	B Coefficient	S.E.	Beta Coefficient		
Constant	6.611	0.505		13.085	<0.001
FSI-10 total score	-0.114	0.010	-0.414	-11.451	<0.001
Age (years)	-0.007	0.002	-0.121	-3.348	0.001
ACQ-6 Total score	-0.097	0.032	-0.106	-2.988	0.003

Table 2 SA. Multiple Linear Regression analysis. Dependent variable: Morisky scale, average values of the two Visits

3.3 Asthma control (ACQ-6)

Adjusted R^2= 0. 737	Unstandardized Coefficients		Standardized Coefficients	t-statistic	p-value
	B Coefficient	S.E.	Beta Coefficient		
Constant	-2.080	0.293		-7.094	<0.001
Baseline ACQ total score	0.828	0.020	0.832	41.138	<0.001
FSI-10 average total score	0.041	0.006	0.135	6.629	<0.001

Table 3 SA. Multiple Linear Regression analysis. Dependent variable: ACQ-6

3.4 Quality of Life (AQLQ)

Adjusted R^2= 0. 723	Unstandardized Coefficients		Standardized Coefficients	t-statistic	p-value
	B Coefficient	S.E.	Beta Coefficient		
Constant	2.818	0.322		8.743	<0.001
Baseline AQLQ total score	-0.802	0.020	-0.820	-39.512	<0.001
FSI-10 average total score	0.054	0.007	0.174	8.304	<0.001

Table 4 SA. Multiple Linear Regression analysis. Dependent variable: AQLQ

3.5 *Lung function*

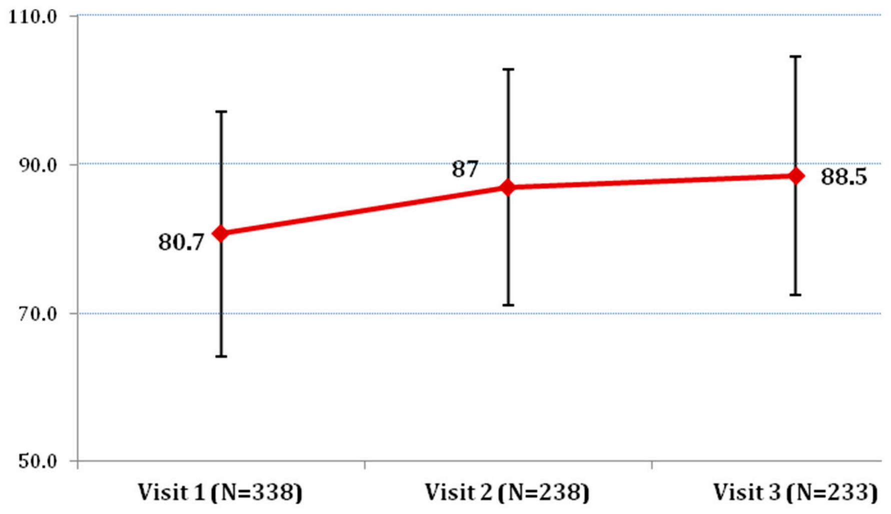

Figure 1 SA. FEV1% predicted /Mean ± SD on the 3 Visits of the study. Base: Observed cases.

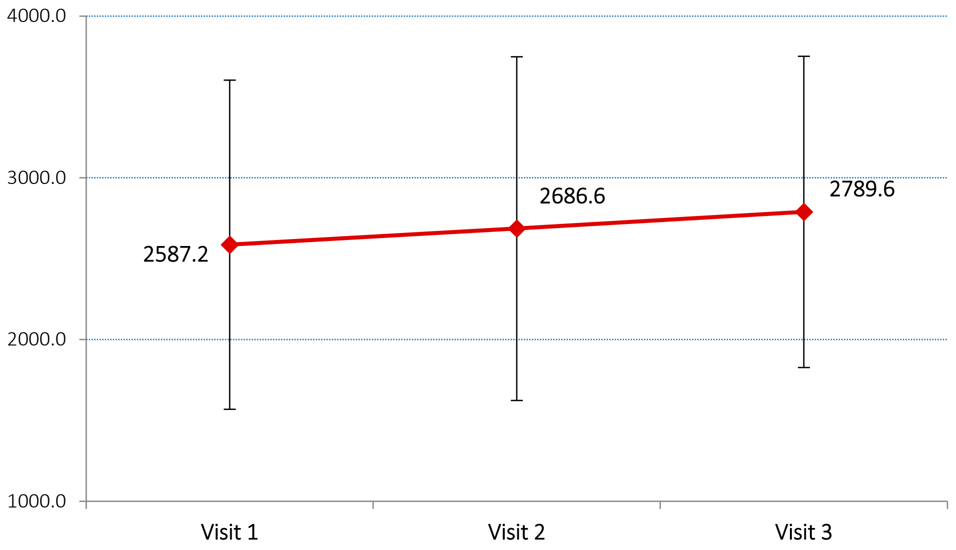

Figure 2SA: FEV1 (mL) Mean ± SD on the 3 Visits of the study. Base: Observed cases.

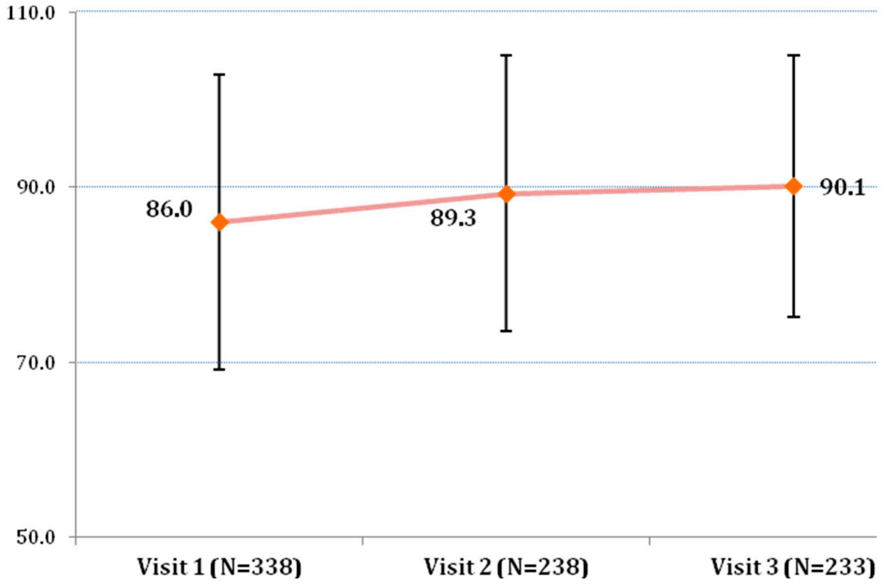

Figure 3 SA. FVC% predicted /Mean ± SD on the 3 Visits of the study. Base: Observed cases.
